# Structural brain abnormalities in postural tachycardia syndrome: A VBM-DARTEL study

**DOI:** 10.3389/fnins.2015.00034

**Published:** 2015-03-17

**Authors:** Satoshi Umeda, Neil A. Harrison, Marcus A. Gray, Christopher J. Mathias, Hugo D. Critchley

**Affiliations:** ^1^Department of Psychology, Keio UniversityTokyo, Japan; ^2^Autonomic Unit, National Hospital for Neurology and Neurosurgery, University College LondonLondon, UK; ^3^Department of Psychiatry, Brighton and Sussex Medical School, University of SussexBrighton, UK; ^4^Sussex Partnership NHS Foundation TrustBrighton, UK; ^5^Sackler Centre for Consciousness Science, University of SussexBrighton, UK; ^6^Centre for Advanced Imaging, The University of QueenslandSt. Lucia, QLD, Australia; ^7^Royal Brisbane and Women's HospitalHerston, QLD, Australia; ^8^Neurovascular Medicine, Imperial College London at St. Mary's HospitalLondon, UK

**Keywords:** autonomic disorders, postural tachycardia syndrome, insula, interoception, salience network, anxiety, depression, VBM-DARTEL

## Abstract

Postural tachycardia syndrome (PoTS), a form of dysautonomia, is characterized by orthostatic intolerance, and is frequently accompanied by a range of symptoms including palpitations, lightheadedness, clouding of thought, blurred vision, fatigue, anxiety, and depression. Although the estimated prevalence of PoTS is approximately 5–10 times as common as the better-known condition orthostatic hypotension, the neural substrates of the syndrome are poorly characterized. In the present study, we used magnetic resonance imaging (MRI) with voxel-based morphometry (VBM) applying the diffeomorphic anatomical registration through exponentiated lie algebra (DARTEL) procedure to examine variation in regional brain structure associated with PoTS. We recruited 11 patients with established PoTS and 23 age-matched normal controls. Group comparison of gray matter volume revealed diminished gray matter volume within the left anterior insula, right middle frontal gyrus and right cingulate gyrus in the PoTS group. We also observed lower white matter volume beneath the precentral gyrus and paracentral lobule, right pre- and post-central gyrus, paracentral lobule and superior frontal gyrus in PoTS patients. Subsequent ROI analyses revealed significant negative correlations between left insula volume and trait anxiety and depression scores. Together, these findings of structural differences, particularly within insular and cingulate components of the salience network, suggest a link between dysregulated physiological reactions arising from compromised central autonomic control (and interoceptive representation) and increased vulnerability to psychiatric symptoms in PoTS patients.

## Introduction

Postural tachycardia syndrome (PoTS) is an increasingly recognized form of dysautonomia, which is characterized by orthostatic intolerance with tachycardia, and accompanied by posture-related autonomic symptoms including palpitations, light-headedness, clouding of thought, and blurred vision. PoTS has also been associated with symptoms of chronic fatigue and high levels of a number of other psychiatric symptoms particularly panic, anxiety and depression (Grubb et al., 2006; Garland et al., 2007; Thieben et al., 2007; Low et al., 2009; Freeman et al., 2011; Mathias et al., 2012). The estimated prevalence of PoTS is at least 0.14%, which is probably about 5–10 times as common as the better characterized condition orthostatic hypotension (Low et al., 2009). The number of published studies of PoTS have grown over recent several years, however most of these studies have examined PoTS from a neurological perspective or focussed on defining the characteristic pattern of peripheral autonomic nervous system dysfunction (Mathias et al., 2012).

From neurophysiological perspective, understanding of central control of autonomic regulation has reached a critical point. Step-changes in our understanding of central autonomic regulation are promised by technical advances and associated insights, as exemplified by the fine-grained data from combination of BOLD fMRI with muscle sympathetic nerve activity (MSNA) recordings (Macefield and Henderson, 2010; James et al., 2013). Within this context, enhanced sympathetic activation is implicated in the pathophysiology of PoTS (Lambert et al., 2008).

From the psychiatric perspective, anxiety and depression symptoms and traits are commonly observed in patients with PoTS (Linzer et al., 1991; Kapoor et al., 1995; Ventura et al., 2001; Kouakam et al., 2002; Mathias et al., 2012). Studies using instruments sensitive to somatic anxiety have also demonstrated abnormally high levels of anxiety and somatosensory hypervigilance in PoTS cohorts (Raj, 2006; Masuki et al., 2007; Raj et al., 2009; Kanjwal et al., 2010). Another study reported that patients with PoTS displayed significantly elevated anxiety sensitivity (which is the fear of anxiety-related sensations) in relation to cardiac symptoms (Taylor and Cox, 1998; Anderson et al., 2014). These observations suggest a link between heightened autonomic reactivity in PoTS patients and their vulnerability to anxiety symptoms (which are intensified by states of uncontrolled autonomic arousal). However, a comprehensive understanding of the neurobiological basis for the increased expression of non-autonomic (psychological) symptoms of PoTS is yet to be fully realized. Here we used neuroimaging of PoTS patients to determine whether there is an explanatory relationship between regional differences in brain structure and general vulnerability to autonomic and psychiatric symptoms.

Past neuroimaging studies examining differences in brain structure and function associated with neurological autonomic disorders have helped characterize the human central autonomic network. For example, patients with pure autonomic failure (PAF), who are unable to generate autonomically-mediated changes in bodily state due to *peripheral* autonomic denervation, have served as an experimental lesion-deficit model to examine contributions of autonomic arousal to cognitive and emotional processes (Mathias and Bannister, 1999; Critchley, 2005). Neuroimaging studies in patients with PAF have demonstrated functional abnormalities within dorsal brainstem, cingulate and insular cortices during effortful behaviors that normally engender increased sympathetic cardiovascular arousal (Critchley et al., 2001). Further, differences in amygdala and insula reactivity to emotional challenges (threat) that typically evoke sympathetic responses in controls have helped demonstrate that higher brain regions involved in motivational and emotional processes are additionally sensitive to the absence of autonomic responses.

In contrast to the “hypo-reactive” syndrome of PAF, there are a number of autonomic disorders including vaso-vagal syncope (VVS: simple or emotional fainting), a common response trait associated with blood and needle phobia that are associated with autonomic “hyper-reactivity.” For example, structural neuroimaging in VVS has suggested that reduced volume within brainstem centers may account for a vulnerability to fainting however structural differences within striatal regions appear more closely tied to fainting frequency, parasympathetic tone and expression of anxiety (Beacher et al., 2009). In contrast, larger amygdala volumes have been reported in individuals with joint hypermobility, a constitutional variant associated with PoTS that is also associated with increased anxiety vulnerability (Eccles et al., 2012). From this perspective, a neuroimaging study of PoTS is strongly motivated to understand and clarify the basis of neurological and psychiatric symptoms experienced by patients with this diagnosis.

In the present study, we used magnetic resonance imaging (MRI) with voxel-based morphometry (VBM) applying the diffeomorphic anatomical registration through exponentiated lie algebra (DARTEL) procedure to investigate whether PoTS is associated with discrete changes in regional brain gray (GM) and white matter (WM) volume. We also administered the State-Trait Anxiety Inventory (STAI), the Panic Disorder Severity Scale (PDSS) and the Beck Depression Inventory-II (BDI-II) to index anxiety, panic and depression symptoms as well as NEO PI-R to measure personality traits according to the five-factor model. These questionnaires were then used to investigate relationships between psychiatric symptom expression and symptom-relevant structural brain differences. Based on earlier findings, we hypothesized that brain regions such as the amygdala, insula and anterior cingulate cortex, that have been implicated in autonomic control, interoceptive representation and emotional behavior will additionally distinguish PoTS patients from healthy controls.

## Methods

### Participants

Eleven patients (9 female; mean age 32.2 years; range 21–50 years) with established diagnoses of PoTS (Freeman et al., 2011; Mathias et al., 2012) and 23 age-matched healthy controls (17 female; mean age 29.0 years; range 18–55 years) participated in this study. At the time of participation, all of the PoTS patients attended a tertiary specialist clinical service for diagnosis and management of autonomic disorders. Each patient had orthostatic intolerance associated with a heart rate increase of 30 beats/min and/or a heart rate that exceeded 120 beats/min within the first 10 min of standing or upright tilt. These patients did not have orthostatic hypotension; rather they showed no change or even a small increase in blood pressure while they were in the upright posture. Seven patients were prescribed medication affecting blood pressure or cardiac responsivity, but were requested to stop taking this on the day of scanning. Among the 11 PoTS patients, five had Ehlers-Danlos Syndrome—hypermobility type/joint hypermobility syndrome (EDS-HT/JHS) (Kanjwal et al., 2010; Mathias et al., 2012), which is frequently observed in PoTS patients. Control participants did not have any history of neurological or psychiatric illness and none was taking prescribed medication. None of the controls had joint hypermobility.

All participants had normal or corrected-to-normal vision. Written informed consent was obtained before inclusion in the study. Experimental work was given ethical approval by the Institute of Neurology/UCLH Joint Research Ethics Committee and the study was performed in accordance with the ethical standards laid down in the 1964 declaration of Helsinki.

### Assessment of psychological and personality characteristics

We used the State-Trait Anxiety Inventory (STAI) (Spielberger and Gorsuch, 1983), the Panic Disorder Severity Scale (PDSS) (Shear et al., 1997), and the Beck Depression Inventory-II (BDI-II) (Beck et al., 1996) to measure anxiety, panic and depressive symptoms respectively. Personality was measured according to the five-factor model (Big Five: extraversion, agreeableness, conscientiousness, neuroticism, and openness-to-experience), using the International Personality Item Pool (IPIP) (Goldberg, 1999) based on the NEO PI-R (Costa and McCrae, 1992). All patients and 12 of the controls completed questionnaires for state and trait anxiety, panic disorder, depression and personality. The PDSS and BDI were completed before MRI scanning and the STAI and IPIP after the scanning session.

### Neuroimaging data acquisition

A Siemens Allegra 3 Tesla scanner was used to acquire a high-resolution anatomical image from each participant at the Wellcome Trust Centre for Neuroimaging, UCL Institute of Neurology. The imaging sequence was as follows: whole brain, 3D MPRAGE, transverse acquisition, phase encoding direction: anterior to posterior, matrix size 256 × 256, repetition time (TR) 7.9 ms, echo time (TE) 2.4 ms, 176 slices, slice thickness 1 mm with no gap, flip angle 15°, voxel size 1 × 1 × 1 mm^3^, field of view (FOV) 256 mm.

### MRI data analysis

We used Statistical Parametric Mapping (SPM8; Wellcome Trust Centre for Neuroimaging, http://www.fil.ion.ucl.ac.uk/spm/) on a Matlab platform to analyse the MRI data. MRI images were first segmented into gray and white matter, and cerebrospinal fluid (CSF) using the unified segmentation module (Ashburner and Friston, 2005). These segmented gray and white matter images were then used to obtain a more accurate inter-subject registration model using DARTEL. This model alternates between computing a group template and warping an individual's tissue probability maps into alignment with this template and ultimately creates an individual flow field of each participant (Ashburner, 2009; Ashburner and Friston, 2009). We then normalized each participant's images into MNI space with the normalized images modulated to ensure that relative gray and white matter volumes were well preserved following spatial normalization. Finally, these images were smoothed with an 8 mm full-width-at-half-maximum Gaussian kernel.

To address the issue of absolute vs. relative threshold masking in SPM we used the masking toolbox (http://www0.cs.ucl.ac.uk/staff/g.ridgway/masking/). This uses an automatic mask-creation strategy to find an optimal threshold to binarise an average image. Morphological group differences in GM and WM volumes were then assessed using the general linear model (GLM) in SPM8. Age and gender were entered into the design matrix as nuisance covariates. To exclude global nuisance effects, total intracranial volumes (TIV) of each participant were also entered into the design matrix as a global calculation. A two sample *t*-test was then conducted to identify between group differences in regional gray and white matter volume. Contrast images were overlaid onto the standard T1 template using MRIcron (http://www.mccauslandcenter.sc.edu/mricro/mricron/). Brain imaging data are reported at an uncorrected statistical thresholds of *p* < 0.001.

For subsequent regions of interest (ROI) analysis, we first produced anatomically defined ROIs using the WFU Pick Atlas (http://fmri.wfubmc.edu/software/PickAtlas/). We next extracted participant specific contrast estimates value (beta weights) for all of the voxels within each mask then performed regression analyses to examine the relationship with personality score.

## Results

### Questionnaire data

Compared to controls, PoTS patients scored higher on measures of state and trait anxiety (STAI state anxiety mean score: PoTS 38.5, controls 30.2, *F*_(1, 22)_ = 6.97, *p* < 0.05; STAI trait anxiety mean score: PoTS 48.2, controls 37.7, *F*_(1, 22)_ = 15.25, *p* < 0.01). Additionally, five of the 11 PoTS patients reported a history of panic attacks. Correspondingly, the PoTS group scored higher for panic symptoms (PDSS mean score: PoTS 2.83, controls 0.58). The PoTS group also had significantly higher depression scores than controls (BDI-II mean score: PoTS 14.83, controls 4.33, *F*_(1, 22)_ = 8.98, *p* < 0.01).

Across measures of personality, PoTS patients scored significantly higher on the dimension of “neuroticism” [*t*_(22)_ = 2.07, *p* < 0.05] and lower on the “openness-to-experience” dimension [*t*_(22)_ = 2.07, *p* < 0.05] (IPIP average percentile estimates: neuroticism, PoTS 64.7, controls 37.0; openness-to-experience, PoTS 46.1, controls 69.0).

### Volumetric data of MRI

**Figure 1:**
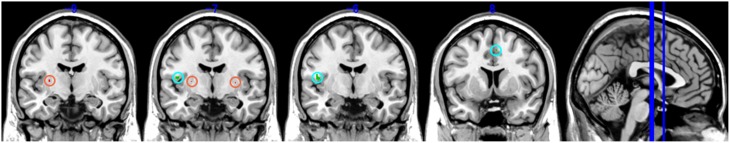
**Brain areas with significant volume reduction of gray matter (GM)**. Green map: Control vs. PoTS, Red map: PoTS vs. Control. From left panel (−9): left putamen, (−7): left insula and bilateral putamen, (−6): left insula, (8): right cingulate gyrus.

PoTS patients showed a similar average total brain volume [GM + WM + CSF (Cerebrospinal fluid)] to controls [1609.6 ml (SD 196.8) vs. 1641.3 ml (SD 220.1), *F*_(1, 32)_ = 0.16, *p* > 0.05]. However, in line with our prior hypothesis PoTS patients showed significant changes in insula volume, specifically a significantly lower left insula gray matter volume compared to controls. Lower gray matter volumes in PoTS were also observed in the right middle frontal gyrus and right cingulate gyrus (Figure 1 green map, uncorrected *p* < 0.001). In contrast, PoTS patients showed an increase in gray matter volume in regions of striatum (bilateral putamen) as well as the right middle temporal gyrus (Figure 1 red map, uncorrected *p* < 0.001).

**Figure 2:**
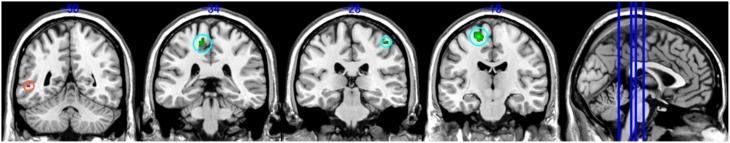
**Brain areas with significant volume reduction of white matter (WM)**. Green map: Control vs. PoTS, Red map: PoTS vs. Control. From left panel (−50): left middle temporal gyrus, (−34): left paracentral lobule, (−28): right postcentral gyrus, (−18): left precentral gyrus.

Our analysis additionally revealed lower WM volume within regions adjacent to precentral gyrus, paracentral lobule, right pre- and post-central gyrus, paracentral lobule and superior frontal gyrus (Figure 2 green map, uncorrected *p* < 0.001). In contrast, PoTS patients showed an increase in white matter volume in the left middle temporal gyrus (Figure 2 red map, uncorrected *p* < 0.001). Of note all findings are reported after controlling for age, gender and total intracranial volume (Table 1).

**Table 1:** **Brain region and peak significance of gray matter (GM) and white matter (WM) volume reduction by group comparison using VBM-DARTEL**.

**Region**	**Extent (k)**	***t*-value**	**MNI coordinates (mm)**		
			**x**	**y**	**z**
**GM: CONTROL vs. PoTS**					
Left insula (BA13)	59	3.7	−47	−6	11
Right middle frontal gyrus (BA6)	23	3.7	26	14	62
Right cingulate gyrus (BA32)	5	3.6	5	8	47
Right cingulate gyrus (BA24)	3	3.6	11	−3	53
**GM: PoTS vs. CONTROL**					
Right putamen	1	3.5	30	−7	4
Left putamen	4	3.5	−29	−9	6
Right middle temporal gyrus (BA37)	1	3.4	42	−63	−6
**WM: CONTROL vs. PoTS**					
Left precentral gyrus	96	6.8	−18	−18	64
Left paracentral lobule	56	4.9	−12	−34	56
Right postcentral gyrus	16	4.0	44	−28	56
Right superior frontal gyrus	5	4.0	8	16	58
Right paracentral lobule	10	3.7	12	−32	56
Right precentral gyrus	5	3.6	30	−18	68
**WM: PoTS vs. CONTROL**					
Left middle temporal gyrus	10	3.8	−52	−50	0

*Table depicts regions with voxel-level significance (uncorrected p < 0.001), the number of voxels in each significant cluster, t-value, and MNI coordinates. GM, gray matter; WM, white matter. Extend threshold was set as 0 voxel for GM contrasts and as 5 voxels for WM contrasts*.

### ROI analyses and the correlation of the personality traits

**Figure 3:**
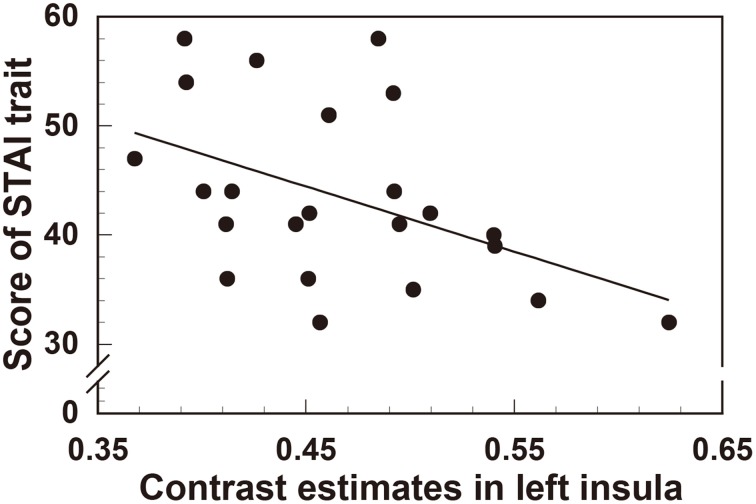
**Significant negative correlation between regional gray matter volume in the left insula and the scores of the STAI (trait anxiety) questionnaire**.

**Figure 4:**
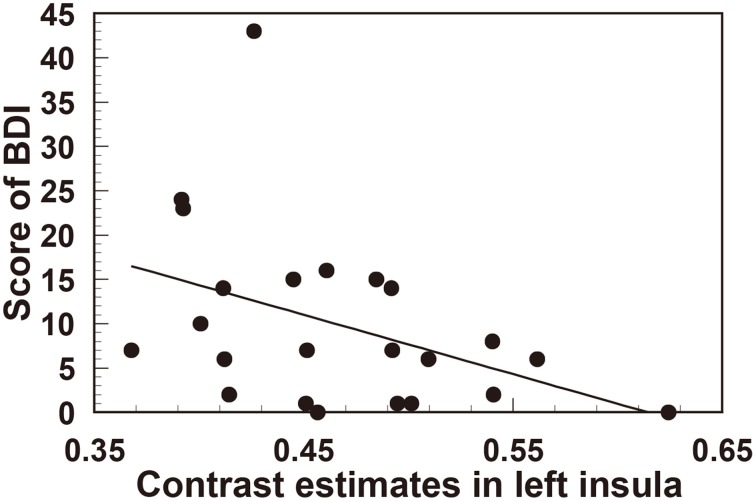
**Significant negative correlation between regional gray matter volume in the left insula and the scores of the BDI (depression) questionnaire**.

In order to quantify the correlation between personality traits and the degree of reduction in left insula (the area with the most significant gray matter volume reduction), we extracted the contrast estimate values from the ROI of left insula, reflecting tissue volume, in all participants (*n* = 23). Interestingly, this revealed a significant negative correlation between left insula volume and both trait anxiety (STAI *r* = −0.463, *t*_(21)_ = 2.39, *p* < 0.05) (Figure 3) and depression score (BDI-II *r* = −0.418, *t*_(21)_ = 2.11, *p* < 0.05) (Figure 4).

## Discussion

The primary aim of this study was to examine variation in regional brain structure associated with PoTS and its relationship to physiological and psychological symptom expression. Our data replicate previous findings of an increase in state anxiety and depression in PoTS patients and additionally demonstrate an increase in neurotic personality traits in this population. These findings provide empirical support for clinical observations of the “emotional style” of PoTS patients which we hypothesized may originate in structural differences within brain regions implicated in autonomic control and emotional arousal. Functional MRI studies have previously shown inter-individual correlations between insula activation and social anxiety and neuroticism scores (Terasawa et al., 2013). It is therefore of interest that we also demonstrate a reduction in left insula gray matter volume in our PoTS group as well as gray matter volume reductions in right cingulate gyri (BA 32, 24). While these regions are implicated in autonomic control and the generation and representation of internal arousal states, they are also viewed as key components within the “salience network” (Menon and Uddin, 2010). To our best knowledge, this is the first study to investigate the anatomical characteristics of the PoTS patients. (e.g., Critchley, 2005; Critchley et al., 2011). Brain areas implicated in sympathetic regulation (Macefield and Henderson, 2010; James et al., 2013) have direct importance to understanding the genesis and expression of PoTS (Lambert et al., 2008). However, these findings might stem from the common specificities of major depression or general anxiety. Previous studies investigating the structural specificities of major depression showed decreased gray matter volume in bilateral insular cortex (Peng et al., 2011). Other study reported larger volume of amygdala and the dorsomedial prefrontal cortex in patients with generalized anxiety disorder (Schienle et al., 2011). Another integrative study examining the shared structural specificity both in depression and anxiety disorders indicated that reduced volume of the rostral-dorsal anterior cingulate gyrus is a generic effect in both disorders (Van Tol et al., 2010). Although some studies are consistent with our finding in terms of the dysfunction within “salience network,” there remains unclear why these regions could be key components in depression or anxiety disorders. Speculatively, the gray matter volume reduction in these areas observed in PoTS patients may underpin an altered sensitivity to detect and predict changes in autonomic bodily arousal. This may translate into abnormal sensitivity to bodily physiological changes and ultimately enhanced vulnerability to anxiety symptoms (Paulus and Stein, 2006, 2010).

This interpretation is also supported by our observation of a significant correlation between the size of insula volume reduction and trait anxiety levels. Moreover, we found that the volume reduction in left insula also predicted depression scores. Our observations of smaller left insula and right anterior cingulate volume suggest a potentially common basis for both the psychological and physiological symptoms observed in PoTS patients, with both originating from dysfunctional integration of the salience network with descending autonomic control. At an operational level, this dysfunction would be expressed as a compromised ability to induce cardiac deceleration to regulate arousal responses to daily challenges; arising from weak interaction between efferent predictions concerning and imprecise interoceptive feedback (Paulus and Stein, 2006, 2010; Critchley et al., 2011). This interpretation is consistent with previously reported positive correlations between the structural and functional integrity of regions including insula and cingulate cortex with individual differences in interoceptive sensitivity and capacity for effective autonomic control (Critchley, 2004; Pollatos et al., 2007).

In addition, it is interesting that we observed differences in WM volume of the PoTS patients bilaterally in the region of primary somatosensory areas (precentral gyri and bilateral paracentral lobules). We suggest that the smaller white mater volumes in these regions reflect limitations in the structural connectivity (and by implication the functional interaction) of primary somatosensory areas with adjacent areas, not least the viscerosensory insula. The anatomical segregation between visceral interoceptive and other somatopic representations is partial: The paracentral lobules are located in the medial parts of the precentral and postcentral gyri and are known to have functions of somatosensory and motor controls of the body, including autonomic functions of defecation and micturition (Fowler, 1999). Interestingly, lesions of the paracentral lobule will engender “stress-sensitive” neurological symptoms including migraine (Schwedt and Chong, 2014). The region also has tight fiber connections to pons and thalamus, so that deficient white matter tracts to these region may also underlie some of the autonomic symptoms manifested by PoTS patients.

In our volumetric analyses, we also discovered significant greater volume in PoTS patients compared to controls within bilateral regions of putamen. The functional contribution of the striatum to autonomic control has been recognized for some decades, and is an important consideration in the understanding of the autonomic failure and dysautonomia associated with multiple system atrophy and related Lewy body diseases (Pastakia et al., 1986). Previous studies suggests that MRI signal reduction in putamen could be an early measure for differentiating multiple system atrophy from pure autonomic failure (Brown et al., 1987). Similar findings also come from radioisotope (PET) studies (Brooks et al., 1990). From an anatomical perspective, the striatum (notably the putamen) receives a major projection from the insula (Saper, 1982). Interestingly, the attenuated volume of another neostriatal region, the caudate nucleus, is implicated in the expression of syncope (also a symptom of PoTS) and its relationship to cardiac parasympathetic tone and anxiety (Beacher et al., 2009). As noted above, PoTS can be characterized by autonomic hyper-reactivity, so that our present results of unbalanced structural features with greater volume of putamen and lower volume of insula and cingulate implicate these areas in the clinical hyper-autonomic symptoms of PoTS patients.

In a related line of research into autonomic function, putamen volumes are reported as significantly larger in patients with obstructive sleep apnea (OSA) (Kumar et al., 2014). A primary characteristic of OSA is impaired autonomic nervous system regulation, reflected especially in excessive sympathetic activity and impaired habituation to autonomic challenges. It is possible to speculate that there is a causal relationship between greater volume in putamen and autonomic hyperactivity. However, we recognize that our findings require replication, not least because the extent volume (the number of voxels) where we detected a significant difference was relatively low. We anticipate that more robust results would be obtained in a future study if the statistical power is improved by increasing the number of participants in both groups.

Some limitations exist in the present study. We recruited 11 patients compared to 23 controls for volumetric comparison. Although our overall findings were based on strict statistical thresholds, the effects reported in this study were nevertheless established on relatively small sample size. Moreover, the results we obtained in the present investigation regarding the relationship between the characteristics of regional brain volume and personality traits were also based on the smaller sample size of controls. While tantalizing, we should await further replication and thus treat the “diagnostic” implications of our findings with caution. Furthermore, the present findings address only characteristics at the structural level, and there remains a need to also compare these to what is known at the levels of functional activation, physiological reactivity, and personality characteristics (Umeda et al., 2009). Clearly, we anticipate some overlap but also, there is likely to be distal consequences of perturbations in the structural integrity of brain regions that contribute to one or more distributed networks. Understanding how structure and function inter-relate and inter-connect is important to gain a comprehensive account of the neurobiology underlying clinical symptoms. One issue not explored in detail within the present study is increasing recognition of an association between PoTS and Ehlers-Danlos Syndrome—hypermobility type/joint hypermobility syndrome (EDS-HT/JHS) (Kanjwal et al., 2010; Mathias et al., 2012). As noted, this condition has itself an established relationship to anxiety disorders, particularly panic disorder (Lang et al., 1998; Hakim and Grahame, 2003; Bulbena et al., 2004; Garcia Campayo et al., 2010). Our present study was underpowered to explore structural differences between the PoTS patients with and without the EDS-HT/JHS, and this is the focus of on-going research (see Eccles et al., 2012). However, the first aim of this study was to identify the characteristics of brain structure across PoTS patients to enhance our understanding of the neural mechanisms of autonomic dysfunction and its correlates with psychiatric symptoms and personality characteristics in the PoTS patients. Differences in the expression of PoTS between patients with and without joint hypermobility merit further investigation. Our present findings provide valuable insight into neural mechanisms underlying the expression of physical and psychological symptoms in an increasingly recognized medical condition.

### Conflict of interest statement

The authors declare that the research was conducted in the absence of any commercial or financial relationships that could be construed as a potential conflict of interest.
